# Role of Cellular Immunity in Cow's Milk Allergy: Pathogenesis, Tolerance Induction, and Beyond

**DOI:** 10.1155/2014/249784

**Published:** 2014-06-09

**Authors:** Juandy Jo, Johan Garssen, Leon Knippels, Elena Sandalova

**Affiliations:** ^1^Program of Immunology, Danone Nutricia Early Life Nutrition, Singapore 138671; ^2^Division of Pharmacology, Utrecht Institute for Pharmaceutical Sciences, Faculty of Science, Utrecht University, Utrecht 3584, The Netherlands; ^3^Department of Immunology, Nutricia Research, Utrecht 3584, The Netherlands

## Abstract

Food allergy is an aberrant immune-mediated reaction against harmless food substances, such as cow's milk proteins. Due to its very early introduction, cow's milk allergy is one of the earliest and most common food allergies. For this reason cow's milk allergy can be recognized as one of the first indications of an aberrant inflammatory response in early life. Classically, cow's milk allergy, as is true for most other allergies as well, is primarily associated with abnormal humoral immune responses, that is, elevation of specific immunoglobulin E levels. There is growing evidence indicating that cellular components of both innate and adaptive immunity play significant roles during the pathogenesis of cow's milk allergy. This is true for the initiation of the allergic phenotype (stimulation and skewing towards sensitization), development and outgrowth of the allergic disease. This review discusses findings pertaining to roles of cellular immunity in allergic inflammation, and tolerance induction against cow's milk proteins. In addition, a possible interaction between immune mechanisms underlying cow's milk allergy and other types of inflammation (infections and noncommunicable diseases) is discussed.

## 1. Introduction

Cow's milk allergy (CMA) incidence characteristically peaks during early childhood and tends to recede later. The prevalence of confirmed CMA is reported to be 0.6–2.5% in preschoolers, 0.3% in older children and teens, and less than 0.5% in adults [1]. However, the self-perceived prevalence is higher: 1–17.5% in preschoolers, 1–13.5% in older children and teens, and 1–4% in adults [1]. This phenomenon, resulting in dietary restriction, may impair the quality of life of both child and family, impede children's growth, and induce unnecessary health care cost [2]. In patients with persistent CMA, the repetitive exposure to cow's milk proteins could result in chronic allergic inflammation accompanied along with anatomical and physiological defects, including eosinophilic gastroenteropathies [3, 4]. In addition, CMA patients, particularly the persistent cases, develop substantial predisposition to respiratory allergies, such as asthma, in their later life, a phenomenon labeled as atopic march [5, 6]. It is noticed that the clinical and lung function outcome in later life is determined by the severity of childhood asthma [7]. Therefore, it is important to understand the CMA pathogenesis in order to effectively prevent and manage the disease and its later life consequences, such as the atopic march.

As reflected by its name, CMA is an immune-mediated aberrant reaction to certain proteins within cow's milk, such as casein (Bos d 8) or beta-lactoglobulin (Bos d 5), which are in principle harmless food ingredients. There are 3 types of inflammatory mechanisms that can mediate CMA: the “acute onset” immunoglobulin E- (IgE-) mediated, the “delayed onset” non-IgE cell-mediated, and the mixed type-mediated allergies. Onset and clinical manifestations of CMA are varied among these clusters [1], hence complicating its proper diagnosis and management. It is important to note that the IgE-mediated CMA typically persists to school age and seemed to be a risk factor for the atopic march [8].

The humoral aspect of CMA immunopathogenesis has been studied quite extensively, partly because there is a significant overlap between mechanisms underlying inflammation due to food allergy and helminth infections. Indeed, it is well known that helminth infections cause T_Helper_2- (T_H_2-) polarized immune responses, hence elevated IgE levels in order to eliminate the parasites [9]. Among the IgE-mediated CMA patients, the early phase of CMA clinical manifestations is due to the cross-linking of surface-bound allergen-specific IgE by allergens that subsequently activate mast cells and basophils to release biologically active substances, such as histamine, interleukin-4 (IL-4), serine proteases, TNF-*α*, and platelet-activating factor [3]. Immunoglobulin free light chains (Ig-fLCs) are proposed to be a proallergic soluble mediator as well. Mechanistically, free *κ* or *λ* light chains were shown to be able to induce murine mast-cell degranulation, causing immediate allergic inflammation [10]. Ig-fLC blockade indeed strongly reduced the allergic skin responses in a murine model of CMA [11]. Interestingly, serum levels of Ig-fLCs in CMA patients were significantly elevated as compared to the ones observed in nonallergic subjects [11] supporting the proallergic role of Ig-fLCs. Elevated levels of Ig-fLCs have been associated with numerous autoimmune diseases as well, for example, systemic lupus erythematosus, multiple sclerosis, and rheumatoid arthritis [12], suggesting that the humoral arm of allergic reaction can also mediate other types of chronic inflammatory diseases.

Nonetheless, immune responses, including allergic reactions, consist of both humoral and cellular components. The availability of proallergic soluble factors is crucially dependent on immune cell activities, hence suggesting the importance of cellular immunity. There is indeed a growing data addressing roles of immune cells, both from innate and adaptive immunity during allergy and upon tolerance induction and maintenance. This review focuses on the roles of cellular immunity in cow's milk-induced inflammation in order to enhance our understanding of CMA immunopathogenesis. In addition, a better understanding of CMA allows scientists to manage (prevent and/or treat) inflammatory diseases due to a significant overlap between mechanisms underlying allergic inflammation and other inflammatory reactions.

## 2. Cellular Immunity during CMA Pathogenesis

**Figure 1:**
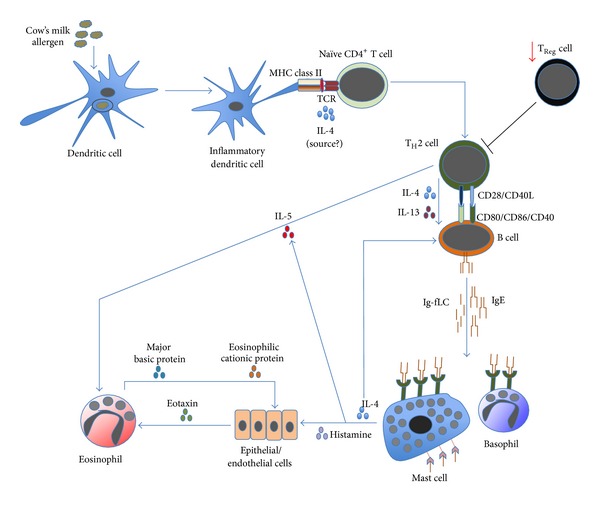
The cascade of allergic inflammation. Allergen's exposure to inflammatory DCs allows these cells to process and to present allergen-derived peptides to naïve CD4^+^ T cells. In the presence of IL-4 (from an unknown source), naïve CD4^+^ T cells differentiate into proallergic T_H_2 cells. Concurrently, it appears that there is an impairment of T_Reg_-cell frequency and/or activity; hence, no suppression is exerted on T_H_2-cell activity. Subsequently, T_H_2 cells will drive B cells, via cell contact as well as IL-4 and IL-13, to undergo immunoglobulin class-switch recombination, in which they eventually produce IgE. Along with the antibody production, B cells also secrete significant amount of *κ* and *λ* Ig-free light chains (Ig-fLCs). IgE and Ig-fLCs will then bind to mast cells and basophils, causing sensitization (not shown). Following subsequent exposure to allergen, cross-linking of surface-bound antibodies occurs (not shown), causing mast cells and basophils to degranulate and release their biologically active substances, including histamine, IL-4, and IL-5. Released IL-4 amplifies the differentiation of T_H_2 and IgE-producing B cells, while released IL-5, also secreted by T_H_2 cells, causes accumulation and activation of eosinophils in the affected tissues. Similarly, histamine activates epithelial or endothelial cells to release eotaxin that also attracts eosinophils into the tissues. Activated eosinophils release active substances, including major basic and eosinophilic cationic proteins that are toxic to the surrounding cells, contributing to further inflammation.

Hereby, cellular components of innate and adaptive immunity are discussed separately. However, it does not imply that these two systems operate independently during allergic inflammation. On contrary, innate and adaptive immune cells influence each other intensively which can contribute to the occurrence of allergy and its clinical characteristics. A key feature is that most allergens, including cow's milk proteins, are sampled, processed, and presented by dendritic cells (DCs) in order to initiate the cascade of cellular and humoral immune reactions leading to allergic inflammation (Figure 1).

### 2.1. Innate Immunity

Three subsets of innate immune cells, that is, tissue mast cells, basophils, and eosinophils, are postulated as the principal effector cells upon allergen exposure. Tissue mast cells and basophils play a pivotal role in IgE-mediated allergy due to their surface expression of high-affinity receptors for IgE (Fc*ε*RI) and their ability to secrete mediators of allergic inflammation after cross-linking by the specific allergens [11, 13]. Hence these cells contribute to the early- (immediate) and late-phase reactions of allergy (2–6 hours after exposure) [3]. In addition, several studies have demonstrated that murine mast cells can be activated by Ig-fLCs to release proallergic mediators as well [10, 11]. Routine assessment assays on mast-cell and basophil activation in humans and mice have been developed and extensively used. Briefly, mast-cell activity in humans can be indirectly measured by the diameter size of the induced wheal after skin prick test (SPT) with milk extract [14], while in mice mast-cell activity can be measured either by the size of cow's milk protein-induced ear-skin swelling or by the elevation of serum levels of mouse mast-cell *β*-chymase/mMCP-1, a specific marker for mucosal mast-cell degranulation [15, 16]. Similarly, basophil activation in humans and mice can be assessed through the measurement of released mediators (e.g., histamine or IL-4) or the upregulation of degranulation-associated cell-surface proteins (CD203c or CD63) [13]. As indicated by the key roles of mast cells and basophils in allergy, wheal diameter and expression levels of CD203c and CD63 on milk-activated basophil were indeed much more pronounced in patients with severe allergy [14].

Mediators released upon mast-cell degranulation, particularly histamine, could stimulate endothelial or epithelial cells to release a potent eosinophil chemoattractant, that is, eotaxin [17]. This causes the infiltration of eosinophils, along with basophils, into inflamed tissues [18]. In addition, mast cells also release IL-5 that attracts eosinophils, prolongs their survival, increases adhesion to endothelial cells, and enhances their effector function [19]. The tissue-infiltrating eosinophils subsequently release highly basic and cytotoxic granule proteins, including major basic protein and eosinophil cationic protein, which are toxic to epithelial and endothelial cells [20, 21] contributing to the late-phase reaction of allergy [3]. This tissue inflammation eventually results in eosinophilic gastroenteropathies [22].

Roles of other innate immune subsets including neutrophils, monocytes, NK, *γδ*, and NK T cells during allergic reaction to cow's milk are unfortunately elusive yet. Neutrophils, *γδ*, and NK T cells were accumulated in the chronically inflamed digestive tissues of CMA patients [23–25], but the actual roles of these innate cells are unknown. It is possible that these cells are accumulated in the inflamed sites mainly because of the elevated levels of inflammatory chemokines during chronic allergic reaction and indirectly activated by the circulating inflammatory cytokines, hence contributing to the chronic inflammatory reactions [3]. Nevertheless, several murine studies suggest that these cells could play key roles during allergy. Neutrophils have been indicated to be important in both sensitization and induction of allergic skin inflammatory reactions as well as mediating alternative mechanisms of anaphylactic reaction [26]. On the other hand, murine tissue *γδ* and invariant NK T cells were suggested to exert regulatory roles to suppress food allergy [27, 28]. Human studies are definitely required to clarify these murine findings. The different cellular elements between human and murine immune systems further complicate the extrapolation of mouse data to the human setting. For example, several studies [29, 30] have demonstrated that while CD1d-restricted invariant NK T cells are abundant in mice but low in human, MR1-restricted mucosal-associated invariant T (MAIT) cells are instead abundant in human but low in mice. Pertaining to CMA, it will be more relevant to study particular immune cells that are enriched in humans.

As a part of professional antigen-presenting cells (APCs), DCs are crucial in order to sample, process, and display antigens to naïve T cells, either to initiate immune responses or to induce immune tolerance [31]. Pertaining to food-derived antigens, roles of DCs in the intestine and associated lymphoid tissues are of particular interest, partly due to the fact that these cells can pick up antigens directly from the intestinal lumen or antigens that have been transported across the intestinal epithelial cells (IECs) [32]. In the healthy gastrointestinal tract, however, commensal bacteria and their products modulate intestinal DCs to be hyporesponsive or tolerant via interaction with the pattern recognition receptors of DCs [32, 33]. In addition, noninflamed healthy IECs are also able to suppress inflammatory DCs while inducing tolerogenic DCs [33]. Taken together, an interaction between gut microbiota, IECs, and intestinal DCs under homeostatic conditions contributes to immune tolerance in the healthy gastrointestinal tract. Of particular interest is the existence of tolerogenic CD103^+^ DCs in murine intestines and mesenteric lymph nodes because they were able to convert naïve CD4^+^ T into FOXP3^+^  T_Regulatory_(T_Reg_) cells via TGF-*β* and retinoic acid [34]. A recent study shows a functional homology between murine CD103^+^ DCs and human CD141^high^ DCs in cross-presenting antigens to CD8^+^ T cells [35], hence eliciting a query of whether human intestinal CD141^high^ DCs can also serve as tolerogenic DCs. Notably in a mouse model of peanut allergy, oral sensitization with peanut extract was accompanied by a shift in intestinal DC subsets, that is, less tolerogenic CD103^+^ DCs but more inflammatory CD11b^+^ DCs [36]. DC-recognition of allergens can be mediated by their C-type lectin receptors, such as DC-SIGN and mannose receptor [37]. Subsequently in the presence of IL-4 (potentially released by allergen-activated innate immune cells), allergen-presenting DCs polarize naïve CD4^+^ T cells into T_H_2 cells, which in turn direct B cells to produce IgE [3]. The important role of DCs for mediating allergic reaction against cow's milk proteins is indeed supported by a finding from the adoptive transfer study of DCs from cow's milk-allergic mice into naïve recipients. Importantly, this DC transfer induced spontaneous production of cow's milk-specific IgE in the naïve mice in the absence of antigen challenge [38]. In summary, inflammatory DCs initiate the allergic reaction by sampling and processing cow's milk allergens and then presenting allergen-derived peptides to CD4^+^ T cells, which will be followed by the activation of proallergic effectors including tissue mast cells, basophils, and eosinophils.

### 2.2. Adaptive Immunity

CD4^+^ T cells serve an important role as the master regulator of adaptive immune responses. Through their plasticity to differentiate into at least proinflammatory T_H_1/T_H_2/T_H_17 or anti-inflammatory T_Reg_ cells, CD4^+^ T cells crucially influence the outcome of inflammatory reactions [39], either resulting as a resolved or persistent inflammation. While T_H_1 and T_H_17 cells are physiologically important to eliminate intracellular and extracellular pathogens, respectively, T_H_2 cells are important for eradicating helminths. However, through secretion of IL-4, IL-5, and IL-13, T_H_2 cells also contribute to the pathogenesis of allergy [3, 39]. Indeed CMA patients exhibited cow's milk protein-specific T_H_2-polarized immune responses in their peripheral blood, that is, high levels of IL-4, IL-5, and IL-13, with low production of T_H_1-cytokine IFN-*γ* [40–44]. Importantly, this T_H_2-cytokine profile was also observed on the duodenum-infiltrating T cells derived from CMA patients upon stimulation with cow's milk proteins [45]. In addition, cow's milk-specific T_H_2-immune responses were observed in murine models of CMA as well [46, 47]. Of note, partly due to many potential allergens within cow's milk, it is still elusive whether there is any difference of T-cell epitopes recognized by CMA patients who developed tolerance and by the ones who developed persistent allergy [48]. Taken together, CMA apparently occurs due to persistent uncontrolled T_H_2-immune responses.

The following question is whether any regulatory mechanism exists to modulate allergy. One possible mechanism is partly attributed to the suppressive role of T_Reg_ cells. These cells can be further classified as thymus-derived, peripherally derived, or* in vitro* induced T_Reg_ cells [49]. However, in order to simplify the nomenclature used in this review, T_Reg_ cells are grouped as one entity. Their suppressive functions can occur through either secretion of inhibitory cytokines (e.g., IL-10 and TGF-*β*), cytolysis, metabolic disruption, or attenuation of DC maturation and/or functionality [50]. It has been shown that a fine balance between T_Reg_ and proallergic T_H_2 cells, including cell frequency and functionality, determines the development of allergy [51]. A noteworthy, supporting evidence of a T_Reg_ suppressive role in allergy came from a clinical study of patients with IPEX (immune dysregulation, polyendocrinopathy, enteropathy, X-linked) syndrome caused by a deletion in a noncoding region of the* FOXP3* gene, the central gene for T_Reg_ differentiation. These patients had defect in T_Reg_ frequency as well as functionality and more importantly exhibited severe food allergic phenotype particularly against cow's milk proteins [52]. IPEX patients also suffer from autoimmune diabetes and/or thyroiditis [53], reiterating that the impairment of T_Reg_ allows many types of inflammation to occur. Indeed CMA patients who had a higher frequency of circulating cow's milk protein-specific T_Reg_ cells exhibited a milder symptom and a favourable prognosis [54]. In addition, lower frequencies of TGF-*β*-producing T cells were observed in the duodenal mucosa of children with food allergy as compared to nonallergic subjects [55, 56]. To summarize, defects in T_Reg_ frequency and functionality partly contribute to CMA pathogenesis.

Despite exogenous antigens, including cow's milk proteins being cross-presented by DCs to initiate CD8^+^ T-cell responses [57], it is unclear whether CD8^+^ T cells play any important role in CMA. It was even reported that upon unspecific stimulation there was a significant difference in the frequency of IFN-*γ*-expressing CD4^+^, but not CD8^+^ T cells, between CMA infants and healthy controls [58]. On the contrary, it is obvious that differentiated B cells (plasma cells) serve an important pathogenic role during allergy. The presence of IL-4 and IL-13 released by T_H_2 cells promotes immunoglobulin class-switch recombination, inducing plasma cells to secrete IgE [3]. Taken together, CMA pathogenesis is attributed to the proallergic activity of cellular components of innate (DCs, tissue mast cells, basophils, and eosinophils) and adaptive immunity (T_H_2 and IgE-producing B cells along with the impaired T_Reg_-cell activity).

## 3. Cellular Immunity upon Tolerance to Cow's Milk Proteins

The majority of infants with CMA spontaneously develop clinical tolerance to cow's milk proteins, that is, no more allergic inflammation by school age [59]. In others words, they will outgrow the allergic disorder to cow's milk. However, the dire consequences due to the dietary restriction before these children outgrow their allergic reactions, the tissue damage due to chronic allergic inflammation, and the potential atopic march in later life serve as an important reminder that CMA needs to be properly managed early on. Several studies have been conducted to discover effective ways to induce tolerance against specific allergens, including allergen-specific immunotherapy. This topic has been extensively reviewed elsewhere [60, 61]. Logically, there are humoral and cellular components of immunity contributing to tolerance. For the humoral arm, within IgE-mediated allergic subjects who later develop tolerance, levels of allergen-specific IgE were reduced in contrast to the increment of allergen-specific IgG4 levels. This alteration is apparently mediated by IL-10 [60]. Mechanistically, IgG4 serves as a blocking antibody via competition with allergens for binding to IgE on the Fc*ε* receptors [62] and as an anti-inflammatory factor due to its dynamic Fab arm exchange resulting as a bispecific antibody with a substantially decreased capacity for cross-linking [63]. In addition, infants who received nondigestible carbohydrates (prebiotics) during the first six months of age had lower incidence of atopic dermatitis, which was linearly associated with lower levels of Ig-fLCs [64]. Taken together, the decreasing levels of allergen-specific specific IgE and/or Ig-fLCs as well as the increasing levels of allergen-specific IgG4 partly contribute to the development of tolerance.

With regard to the cellular immunity, the tolerance mechanism is essentially contributed by two primary mechanisms, that is, (1) the suppression of proallergic innate effectors as well as (2) the upregulation of T_Reg_-cell regulatory activity. Arguably, the latter mechanism is the principal way to induce and maintain tolerance to allergens because it also affects the former mechanism in disease progression. Moreover, functional T_Reg_ cells could contribute to T-cell anergy, that is, a tolerance mechanism in which the lymphocyte is intrinsically functionally inactivated following an antigen encounter but remains alive [65]. Findings of spontaneous and treatment-induced tolerance against cow's milk allergens are discussed together because both approaches, arguably, follow similar immune mechanism.

### 3.1. Inhibition of Proallergic Innate Effector Cells

During tolerance development, proallergic innate effectors could undergo rapid desensitization against allergens, causing them to be less likely to release inflammatory factors [60]. One probable mechanism is due to the presence of allergen-specific IgG4, as mentioned briefly above. Indeed, it has been demonstrated that basophils from CMA children who developed clinical tolerance were significantly less responsive to the allergen [14, 66]. Interestingly, the reduced responsiveness of basophils was partially due to an inhibitory factor present in serum probably allergen-specific IgG4 [66]. It is also known that the secreted anti-inflammatory IL-10 cytokine reduced the release of proinflammatory cytokines by mast cells [67] and suppressed the activity of eosinophils [68]. In addition, a study using the CMA mouse model demonstrated that the synbiotic (prebiotics + probiotics) treatment reduced the anaphylaxis score, in which the induced tolerance was associated with a smaller ear-skin swelling and a lower level of mMCP-1 [69]. Taken together, these findings indicate that tolerance to cow's milk allergens is associated with the suppression of proallergic innate effectors' activity.

### 3.2. Upregulation of T_Reg_-Cell Functionality

Mechanistically, functional allergen-specific T_Reg_ cells can attenuate allergic responses through (1) suppression of mast cells, basophils, and eosinophils; (2) suppression of inflammatory DCs and induction of tolerogenic DCs; (3) suppression of allergen-specific T_H_2 cells, hence contributing to T-cell anergy; and (4) early induction of IgG4 and late reduction of IgE production [60]. All of these mechanisms can be mediated through secretion of IL-10 and TGF-*β* or through cell contact-dependent suppression [60]. Indeed, the increment of frequency and* in vitro* suppressive capacity of T_Reg_ cells were correlated with the clinical tolerance in children who outgrown CMA [70]. Furthermore, by treating a CMA mouse model with various kinds of treatments, including dietary long-chain n-3 polyunsaturated fatty acids, prebiotics,* Bifidobacterium breve* M-16V (probiotics), synbiotics (nondigestible carbohydrates +* B. breve*), or cow's milk protein-derived peptide, a linear correlation of T_Reg_-cell frequency and activity with the CMA suppression is observed [69, 71–74]. It is noteworthy to confirm whether CMA children who develop tolerance after particular immunotherapy will also exhibit increasing T_Reg_-cell frequency and functionality. To summarize, T_Reg_ cells appear to play important regulatory roles upon tolerance to cow's milk allergens.

## 4. Mechanistic Interaction among CMA and Other Inflammatory Reactions: A Broader Perspective

The current findings indicate that CMA, as most other food allergies, is characterized by T_H_2-polarized immune responses accompanied by the impairment of T_Reg_ cells (Table 1). As briefly discussed above, CD4^+^ T cells are heterogeneous due to their ability to differentiate into T_H_1, T_H_2, T_H_9, T_H_17, T_H_22, Tfollicular helper (Tfh), or T_Reg_ cell, hallmarked by different lineage-specifying transcription factors and different signature cytokines [75]. For example, T_H_1 cells express T-bet and secrete IFN-*γ*, T_H_2 cells express GATA3 and secrete IL-4, and T_H_17 cells express ROR*γ*t and secrete IL-17, while T_Reg_ cells express Foxp3 and secrete IL-10 and TGF-*β*. It was originally proposed that each subset of CD4^+^ T cells permanently retains its differentiated identity, resulting as nonoverlapping distinct subsets [75]. However, it is clear now that the differentiation process is dynamic instead, particularly during chronic inflammation* in vivo *[76]. This allows a particular differentiated subset of CD4^+^ T cells to secrete signature cytokines that belong to other subsets or even to further convert into other subsets. For example, it has been shown that T cells derived from chronic allergic asthma patients coexpressed and coproduced both T_H_2 and T_H_17 transcription factors and cytokines [77]. In addition, atopic dermatitis patients predominantly displayed T_H_2-immune responses with a T_H_17 component at the acute phase of the disease, which often converted into T_H_1-immune responses at the chronic stage [78]. Thus, it incites us to wonder whether T_H_2-polarized immune responses in CMA could also exhibit or even convert to T_H_1- or T_H_17-immune responses in minority group of patients who never outgrow their CMA.

Next, the current consensus of food allergy occurs due to the imbalance between T_H_2 and T_Reg_ cells that intrigues us to speculate whether inflammation of CMA affects other types of inflammatory reactions (infection as well as chronic inflammatory noncommunicable diseases/NCDs) and vice versa. The published data does not allow a definite conclusion to be constructed yet; nonetheless it provides some hints that permit various speculations to be made. First, although there is no prospective study following children with food allergy to determine whether they have a lower predilection to suffer from helminth infection, CMA infants with the elevated T_H_2-polarized immune responses should be more protected against helminths. A supporting finding came from a population study in Cameroon which demonstrated that subjects with elevated IL-5 cytokine indeed had reduced reinfection rates with* Ascaris lumbricoides* and* Trichuris trichiura *[79], supporting the importance of T_H_2-immune responses against helminths. On the other hand, helminth infections could induce activation of T_Reg_ cells, resulting in IL-10 and IgG4 production, hence attenuating T_H_2-immune responses [9] and thus may modulate allergic inflammation. Interestingly, a murine study showed that infection with intestinal helminths (*Heligmosomoides polygyrus*) prior to sensitization and challenge with peanut extract* per oral* indeed significantly reduced peanut-specific IgE levels and diminished systemic anaphylactic symptoms via IL-10 production [80]. A study on infants living in areas endemic for helminth infections also suggested that despite potent T_H_2-responses being observed early in life it did not translate into a higher SPT reactivity to various allergens at 4 years of age [81]. Arguably, prior exposure to helminths might reduce allergy incidence to cow's milk proteins as well.

Second, the control and elimination of intracellular pathogens require the activation of T_H_1-immune responses. Since IL-4 suppresses IFN-*γ* gene transcription, that is, T_H_2 cytokine suppresses T_H_1-functionality [82], it is plausible to assume that allergic infants might be more susceptible to be infected with intracellular pathogens. Interestingly, a prospective birth cohort study in The Netherlands, PIAMA (*n* = 4,146), demonstrated an association between children having risk factors for allergy (i.e., having allergic parents and attending child care or having older siblings) with a higher risk of suffering lower respiratory tract infections in the first year of life [83]. However, it is still elusive whether CMA infants are more susceptible than healthy infants to develop infections in the gastrointestinal tract, respiratory tract, or skin. Next, gastrointestinal infection with intracellular pathogens can cause enteral inflammation along with the disruption of the intestinal flora. This perturbs homeostasis between host immunity and gut antigens, which may represent the critical determinant in the development of food allergy, including CMA [84]. Indeed, there is a case report demonstrating a Japanese infant who developed CMA associated with enterotoxigenic* Escherichia coli* and methicillin-resistant* Staphylococcus aureus* infections [85]. In addition, another study of Japanese newborns who underwent small intestine surgery and received antibiotics due to symptoms resembling postoperative infection showed that 9 out of 30 subjects subsequently developed CMA [86]. Importantly, within a subset of patients who received prophylactic probiotics, most of the patients (~98%) did not suffer from CMA [86], suggesting that restoration and maintenance of gastrointestinal immune tolerance is imperative in order to prevent allergy to food antigens. Therefore, gastrointestinal infections that incite enteral inflammation may represent an important risk factor to develop CMA.

Third, consistent with the fact that allergy is the most common and earliest-onset of inflammatory NCDs [87], it is important to understand how the immune mechanisms underlying food allergy interact with the ones constituting other NCDs, including other types of allergy, metabolic diseases, autoimmunity, and cancer. It is noteworthy to mention that there are common risk factors for most NCDs, that is, diet patterns, microbial patterns, behaviour, and environmental pollutants [87]. These common risks may initiate similar alterations within the immune system to cause many NCDs, arguably through the impairment of T_Reg_ cells. T_Reg_-cell defect causes uncontrolled inflammation [50], which in turn underlies most of NCDs [87]. For example, a prospective mother-child study conducted in Germany, LINA (*n* = 629), demonstrated a clear correlation between history of maternal exposure to tobacco smoke and lower T_Reg_-cell frequencies in maternal and cord blood, as well as a higher risk for those children to develop atopic dermatitis within the first 3 years of life [88]. In addition, reduction of T_Reg_ cells has been linked to the dysregulated inflammation of other NCDs, such as obesity and insulin resistance [89]. It is arguable that the common defect of immune regulation may cause several NCDs to occur concurrently, though the responsible mechanism still needs to be confirmed. Nonetheless, it is interesting to quote recent data from the National Health and Nutrition Examination Survey, demonstrating that the US children and adolescents who were obese indeed had higher levels of total IgE and C-reactive proteins as well as higher incidences of food allergy [90]. Taken together, similar impairment in the immune mechanism that causes inflammation may mediate occurrence of many NCDs.

## 5. Conclusion

Hereby findings pertaining to roles of cellular immunity upon allergy as well as tolerance toward cow's milk proteins were discussed. The activation of proallergic innate (inflammatory DCs, tissue mast cells, basophils, and eosinophils) and adaptive effectors (T_H_2 and IgE-producing B cells) and the suppression of T_Reg_ cells collectively contribute to the pathogenesis of CMA. On the other hand, tolerance against cow's milk allergens is contributed by the activation of T_Reg_ cells and the suppression of proallergic effectors mentioned above. A possibility that immune mechanisms underlying CMA interact significantly with the mechanisms underlying other types of inflammation (infections or NCDs) has been raised as well, suggesting that a proper management of CMA may positively contribute to a better control of systemic inflammation.

## Figures and Tables

**Table 1:** Roles of cellular immunity in cow's milk allergy.

Type	Cell	Role in cow's milk allergy
Innate cells	Tissue mast cells	Act as **key effectors** during allergy. Upon Ig-E or Ig-fLC cross-linking with allergen, 3 classes of biologically active product are secreted [15] as follows. (1) Prestored cytoplasmic granules: (a) biogenic amines (e.g., histamine), (b) serglycin proteoglycans (e.g., heparin and chondroitin sulphate), (c) serine proteases (tryptases, chymases, and carboxypeptidases), (d) some cytokines (e.g., TNF-*α* and VEGFA). (2) Lipid-derived mediators (prostaglandins, leukotriene B4, cysteinyl leukotrienes, and platelet-activating factors). (3) Newly synthesized factors (cytokines, chemokines, and growth factors).
	Basophils	Act as **key effectors** during allergy. Similar to mast cells, upon cross-linkage of IgE, 3 types of mediators can be released [13] as follows. (1) Preformed, immediately released (e.g., histamine). (2) Newly synthesized, immediately released (phospholipid metabolites including leukotriene C4). (3) Newly synthesized, slowly released (cytokines including IL-4).
	Eosinophils	Act as **key effectors** during allergy. Upon activation with cytokine (e.g., IL-5), highly basic and cytotoxic granule proteins are secreted [21] as follows. (1) Major basic protein/MBP and MBP2. (2) Eosinophilic cationic protein/ECP. (3) Eosinophilic peroxidase/EPX. (4) Eosinophil-derived neurotoxin/EDN.
	Inflammatory dendritic cells/DCs	Act as **the initiator of ** **T**_**H**_ **2-cell response** during allergy. Inflammatory DCs uptake and process allergens, subsequently presenting allergen-derived peptides to naïve CD4^+^ T cells. In the presence of IL-4, DCs polarizing naïve CD4^+^ T become T_H_2 cells.
	Other innate cells (neutrophils, NK, MAIT, and *γδ* T cells)	Unknown roles.

Adaptive cells	CD4^+^ T_H_2 cells	Act as **the driver** of allergic inflammation. Through cell-contact and cytokines (IL-4 and IL-13), T_H_2 cells promote immunoglobulin class-switch recombination in B cells to drive IgE production.
	CD4^+^ T_Reg_ cells	Act as **the suppressor** of allergic inflammation, via [60] the following. (1) Suppression of tissue mast cells, basophils, and eosinophils. (2) Suppression of inflammatory DCs and induction of tolerogenic DCs. (3) Suppression of allergen-specific T_H_2 cells. (4) Early induction of IgG4 and late decrease in IgE.
	B cells	Act as **the codriver** of allergic inflammation along with T_H_2 cells by secreting IgE and Ig-fLCs.
	Other CD4^+^ and CD8^+^ T cells	Unknown roles.
